# Partial Anterior Cruciate Ligament Ruptures: Advantages by Intraligament Autologous Conditioned Plasma Injection and Healing Response Technique—Midterm Outcome Evaluation

**DOI:** 10.1155/2018/3204869

**Published:** 2018-07-25

**Authors:** Matthias Koch, Felix Mayr, Leonard Achenbach, Werner Krutsch, Siegmund Lang, Franz Hilber, Johannes Weber, Christian G. Pfeifer, Rebecca Woehl, Jürgen Eichhorn, Johannes Zellner, Michael Nerlich, Peter Angele

**Affiliations:** ^1^University Medical Centre Regensburg, Department of Trauma Surgery, Franz Josef Strauss Allee 11, 93042 Regensburg, Germany; ^2^Sporthopaedicum Straubing, Regensburg, Hildegard von Bingen Strasse 1, 93053 Regensburg, Germany

## Abstract

The historical treatment options for partial anterior cruciate ligament (ACL) ruptures were conservative therapy or ACL reconstruction by injured bundle or entire ACL replacement. In awareness of the regenerative potential of biologic agents such as mesenchymal stem cells or platelet rich plasma (PRP), the healing response technique was developed to preserve the injured ACL with better outcomes than the conservative therapy. Further improvement of this technique seems to be obtained by the additional application of PRP products. Thus, the aim of this study was to evaluate the midterm outcome after intraligament autologous conditioned plasma (ACP) by a clinical, scoring, and functional performance assessment. 42 patients were evaluated in this study. The failure rate was 9.5%. Outcome evaluation showed good to excellent results. The scores were IKDC subjective 83.2 (SD 14.5), Lysholm 85.5 (SD 15.5), Tegner 4.7 (SD 1.7), and Cincinnati 85.4 (SD 15.5) after a mean follow-up of 33 months. Clinical examination showed stable Lachman test, negative pivot shift phenomenon, and a significant reduction in AP-laxity compared to preoperative status (rolimeter preoperative: 1.9 (SD1.4); postoperative 0.6 (SD1.8), p=0.001) in all patients. Functional performance testing showed no significant differences between the injured and healthy side. Return to sport was achieved after a mean of 5.8 months (SD 3.6) in 71.1% of the included patients. In summary, this new treatment option revealed in midterm follow-up promising results to treat partial ACL lesions with a reduced need for conversion to ACL reconstruction and with a high percentage of return to preinjury sport activity.

## 1. Introduction

Partial anterior cruciate ligament (ACL) ruptures are a challenging condition for orthopaedic surgeons. The prevalence ranges up to 28% [1–3]. Diagnosis and management of this type of ACL lesions are still under discussion [3, 4]. A reliable assessment of the extension of injury of partial ACL tears usually requires multiple findings by clinical examination, MRI examination, and almost confirming arthroscopically examination [3]. The therapeutic options for the treatment of partial ACL tears historically range from conservative treatment up to partial reconstruction in terms of a bundle augmentation or complete ACL reconstruction according to the injured bundles [1, 3]. The choice of therapy depends on the physical demands of the patients, clinical proven instability, location, and amount of tear as well as concomitant injuries [1]. However, there are many limitations for these therapeutic options. Conservative therapy of partial ACL tears is associated with a high failure rate and often consecutive complete ACL rupture, followed by ACL reconstruction. Initial ACL reconstruction is reported to be potentially associated with diminished proprioception, postoperative muscular weakness, no fully restoration of normal kinematics, donor site morbidity, and possible premature osteoarthritis [4]. Thus, ACL preserving techniques have to be favoured.

Regarding the awareness on the role of biologic agents, such as growth factors and stem cells, in promoting tissue healing further therapeutic options for the therapy of partial ACL tears were developed. In the most known “healing response technique” introduced by Steadman et al. bone marrow stimulation by microfracturing of the lateral fossa intercondylaris of the femur is performed to obtain a clot formation near to the femoral insertion of the torn ACL [4–7]. However, the clinical results are still under discussion. While Steadman et al. showed promising results [5, 6], Wasmaier et al. stated contrary results with no beneficial effects in comparison to the conservative approaches regarding clinical scores, rate of revision surgery, or joint laxity [4, 8].

However, the “healing response technique” can auspiciously be upgraded by the additional application of platelet rich plasma (PRP) [1, 9]. PRP products are already in clinical practice for many orthopaedic disorders, such as osteoarthritis, tendinopathies, or ligament injuries [1, 10–12]. Preclinical studies demonstrated the qualities of PRP in the regulation of the articular environment, exerting a positive metabolic modulation on all joint tissues and promoting tissue healing. Furthermore, PRP was associated with beneficial effects in stimulating fibroblasts proliferation, collagen fibres deposition, and reducing catabolic distress, when applied to ACL-derived tenocytes [13, 14]. Also in vivo studies emphasise the beneficial effect of PRP augmentation, which provide better histological appearance and superior biomechanical properties [15, 16]. So, according to Andriolo et al. there is a strong rationale for the use of PRP to improve ACL healing [1, 17].

Thus, the aim of this study was to evaluate the midterm outcome of patients having partial ACL tears treated by intraligament injection of autologous conditioned plasma (ACP) (Arthrex) and healing response technique according to both, functional, and clinical criteria.

## 2. Material and Methods

The study was approved by the University Hospital Ethic Committee and Scientific Board, and written consent was collected for each patient.

### 2.1. Patient Selection

Patients had been elucidated concerning intraoperative assessment of the ACL rupture and intraoperative decision concerning the treatment (ACL reconstruction versus healing response technique and intraligament ACP injection) in detail before. During regular arthroscopy, patients were screened for partial ACL rupture, while partial ACL rupture was defined as “a partial rupture of the anteromedial or posterolateral or partial rupture of both ACL bundles” and the presence of exclusion criteria. The degree of partial ACL rupture was classified according to a previously introduced ACL grading system [1], which describes the amount of injured ACL tissue in five increasing steps (see Table 1).

**Figure 1:**
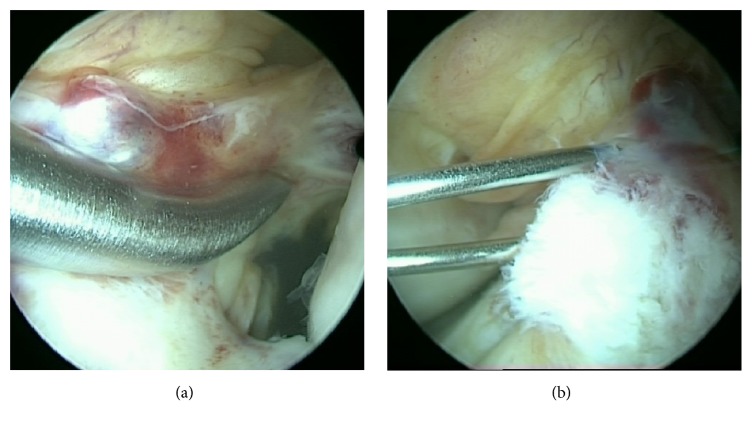
(a) Healing response technique and (b) intraligament ACP injection in a partial ACL rupture grade 3.

In case of a confirmed diagnosis intraligament ACP injection and healing response technique were performed as previously described by Koch et al. [1] (see Figure 1).

Inclusion criteria were defined as patients aged > 18 years and affected by partial ACL rupture according to the definition above.

Exclusion criteria were defined as previously described [1]:

(1) complete tear of at least one bundle of the ACL;

(2) previous or concurrent major cartilage procedures and meniscus replacement;

(3) previous or concurrent ligament reconstruction in the index knee joint;

(4) presence of rheumatic diseases or chronic inflammatory arthropathy;

(5) therapeutic anticoagulation;

(6) further other surgical procedure in the index knee joint within 12 months from the present treatment.

### 2.2. Surgical Procedure and Rehabilitation

Partial ACL rupture was confirmed during diagnostic arthroscopy as described above. Intraligament ACP application into the distal ACL stump and healing response technique at the lateral femoral fossa intercondylaris as well as postoperative care were performed as previously described by Koch et al. [1].

After removal of the arthroscopic fluid both procedures were realised as the last procedure during surgery. No drains were inserted. For postoperative rehabilitation the index knee was immobilized in a 20° flexed splint and partial load bearing (10 kg body weight) was followed for 1-2 weeks. During this period passive range of motion with 0-20-60° trained by the physiotherapist was allowed. Full extension had to be avoided to reduce tension on the ACL during the initial healing period. Full weight bearing started in the third week. For additional external stabilization an ACL brace with a limited range of motion (lack of 10° extension to 90° flexion) during the day and a 20° flexed immobilizing splint overnight were applied. Propriozeption was trained by physiotherapy. After 6 weeks training intensity increased to active assisted and active muscle strengthening.

### 2.3. Patient Evaluation and Follow-Up

For retrospective midterm outcome evaluation at follow-up, all patients were screened for the presence of exclusion and failure criteria. Outcome was assessed using the following items:

1. patients' history:
2. patient satisfaction, time to return to sport (RTS), and postoperative complications;
3. clinical scores:
4. IKDC subjective/objective, Lysholm, Tegner, Cincinnati Scores, and the Marx Activity Scale;
5. clinical examination:
6. rolimeter assessment and clinical stability testing (Lachman test, pivot shift test);
7. functional performance tests:
8. drop-jump test, side-hop test, one- and two-leg stability test, and quick-feet test.

Failure was defined as

1. persisting knee instability with general indication for ACL reconstruction;
2. side to side difference > 4 mm in rolimeter analysis of the knee joint;
3. persisting positive pivot shift test or no firm endpoint in Lachman-testing in the follow-up evaluation.

### 2.4. Statistical Analysis

Statistical analysis was performed using the SPSS software version 23.0 (SPSS, Chicago, IL, USA) to determine relationships between the different variables. To determine whether data followed a Gaussian distribution a Kolmogorov-Smirnov-test was performed. Due to nonnormal distributed data Wilcoxon signed-rank test was used for quantitative data analysis. The significance level was set at p ≤ 0.05.

## 3. Results

In total 42 patients after intraligament ACP application and healing response in partial ACL ruptures were reviewed in this retrospective study. Four patients had to be excluded due to the exclusion criteria. One patient of those had an Achilles tendon rupture during follow-up period, so no usable follow-up assessment was available. One patient developed a symptomatic progressing cartilage defect and received a reoperation during the follow-up period. Two patients had a complete rupture in the index knee after ACP treatment that required ACL reconstruction. At the end, n = 38 patients aged between 18 and 70 years with a mean age of 42.8 years (SD 13.5 years) were included. The mean follow-up after index surgery was 33.0 months (SD 17.4 months). Concerning the gender distribution, 17 (44.7%) male and 21 (55.3%) female patients were treated in context of this study. Overall, in n = 25 cases (65.8%) the right knee and in n = 13 cases (34.2%) the left knee were injured. In n = 30 (78.9%) of the affected patients the dominant side was concerned. Concomitant injuries of the knee joint, such as meniscus lesions or injury of the collateral ligaments, were registered in 55.3% (see Table 2).

In 84.2% of the cases, patients injured themselves by a sport associated trauma (62.5% ski; 15.6% football; 6.25% volleyball; 3.1% mountain biking; 3.1% kung-fu). However, just the minority of the patients (2.6%) performed professional sport (see Table 2).

After index surgery, patients returned to sport after a mean period of 5.8 months (SD 3.6 months). Full sportive activity level was regained by 71.1% of the included patients during the follow-up period. Overall, all patients subjectively regained in mean 85.8% (SD 19.0%) of their preinjury sportive activity level (see Table 3).

Concerning the clinical outcome evaluation, a firm endpoint in the ACL stability testing and no positive pivot shift glide test was documented for all assessed patients. Femorotibial translation was quantitatively analysed by rolimeter testing and significant reduction (preoperative: 1.9 mm (SD 1.4 mm) versus postoperative at the latest FU: 0.6 mm (SD 1.8 mm)) was registered after combined intraligament ACP application and healing response technique (p = 0.001) (see Table 4).

Activity scores, such as IKDC subjective score, Lysholm score, Tegner activity score, Cincinnati score, Marx scale, and IKDC objective score, revealed almost full recovery of the functional activity level at the latest follow-up (see Table 5).

For objective outcome evaluation established functional performance tests, like the drop-jump test, side-hop test, and one- and two-leg stability test as well as quick-feet test, were performed. The comparison of the index versus the healthy side in the drop jump as well as the one-leg-stability test showed no significant differences. Overall, good to excellent results were achieved (see Table 6).

## 4. Discussion

The current study showed for the first time midterm outcome results of patients after an ACL preserving procedure in terms of intraligament ACP application and healing response in partial ACL ruptures evaluated by functional performance tests. Additionally it presents a big cohort of patients after partial ACL rupture treated with intraligament ACP application and healing response assessed by clinical tests and clinical scores with the longest follow-up currently available. In total, 42 patients with a mean follow-up of 33 months (SD 17.4 months) were initially reviewed in this study. However, 4 out of 42 (9.5%) patients had to be excluded as a failure; whereas one had an Achilles tendon injury during follow-up, so the follow-up assessment could not be completed. One patient received an operative cartilage therapy during the follow-up period because of a symptomatic progressing cartilage lesion. A consecutive complete ACL rupture was diagnosed and treated with entire ACL replacement in two patients during follow-up period. Previous studies evaluating the effect of PRP products in partial ACL therapy are limited to a mean follow-up of at least up to 25.1 months (SD 10.0 months) [1, 18]. Overall, a satisfactory outcome after intraligament ACP application and healing response was detected with a low failure rate of 9.5% and a high percentage of return to sport activity (85.8%, SD 19%) as measured by the preinjury sport level.

The therapy of partial ACL ruptures is still challenging. Historically, the treatment options ranged from conservative therapy of partial ACL tears to reconstruction of the entire ACL. The conservative treatment was used to be associated with a high failure rate because of the low endogenous regeneration potential of the ACL, based on the weak blood supply, and high rate of consecutive complete ACL ruptures based on the persistent joint instability [4]. Due to this fact, in the past there was a trend to reconstruct the entire ACL. However, regarding the comorbidities, such as donor site morbidity, loose of natural anatomy, physiology, intrinsic cell population, or proprioception after entire ACL reconstruction [4, 19], entire ACL replacement was found to be an overtreatment for partial ACL lesions. ACL “augmentation” in terms of a selective replacement of the injured ACL bundle was propagandised to preserve healthy parts of the ACL [1, 2, 4].

Based on the purpose to preserve healthy ACL tissue and regarding the increasing knowledge about stimulation of endogenous regenerative potential by the use of biologic agents, such as growths factors and stem cells, Steadman et al. developed the healing response technique to promote ACL tissue healing [5, 6]. By trephination of the fossa intercondylaris of the lateral femoral condyle next to the ACL insertion there was an inflow of growths factors and mesenchymal stem cells out of the femoral condyle into the ACL defect site stimulating tissue regeneration and scarring [1, 4–7]. Steadman et al. showed promising clinical results after performing this healing response technique in proximal ACL tears of skeletally immature athletes and older active patients [5, 6]. Overall, it was concluded that this technique is a promising tool for the treatment of very proximal ACL ruptures in young and middle aged patients [1, 5–7]. However, Wasmaier et al., reviewing clinical and radiological long-term results, were not able to comprehend the promising effect of the healing response technique in 30 young patients in comparison to a conservative treatment of proximal ACL tears [1, 4, 7, 8]. ACL insufficiency required subsequent ACL reconstruction in 36% of the evaluated patients compared to 56% of the conservative treated patients after ACL rupture [1, 8]. Also, the remaining patients (64%) showed no better outcome results than the conservative treated patients [1, 8].

However, in the awareness of the regenerative potential of platelet rich plasma (PRP) products, in the present study the healing response technique according to Steadman et al. was improved by the additional application of a commercially available PRP product, ACP.

PRP products are obtained by concentration from peripheral blood and have already been successfully applied in the treatment of many orthopaedic disorders, such as osteoarthritis, tendinopathies, or ligament injuries [1, 10–12]. As such a biologic agent, the PRP product ACP is able to mediate the tissue regeneration by influencing the inflammatory and remodelling process [4, 20]. This tissue healing enhancing effect is also based on the involvement of the platelets in the joint homeostasis, aggregation, and clot formation steps by the release of several growths factors [4, 21]. In this context, some in vitro studies showed beneficial effects of PRP on the stimulation of fibroblast proliferation, collagen fibre deposition, and reduction of catabolic distress, when applied to ACL-derived tenocytes [1, 13, 14]. Consequently, there are also some in vivo and clinical studies promoting the use of PRP products for enhancing ACL healing [1, 7, 9, 15, 16, 18, 22, 23]. Thus, there is a strong rationale to combine the beneficial effect of ACP with the regenerative effect of the healing response technique. Regarding the current literature most ACL tears are located in the midsubstance area of the ACL especially in younger patients [24]. Assuming a decreasing effect of the healing response technique dependent on an increasing distance from the femoral ACL insertion to the lesion site in midsubstance lesions, an increasing impact of the ACP application for ACL regeneration has to be postulated. So, by application into the distal ACL stump a local depot of PRP can be placed next to the lesion site to focus the regenerative effect on the target area and to provide a longer release of growth factors by reducing a wash out phenomenon, which needs to be considered after intra-articular application.

Overall, there are just few clinical studies currently available, evaluating the effect of PRP on the ACL healing in partial ACL tears. Seijas et al. showed positive results in football players after application of a PRP product into the proximal and distal ACL stump without healing response after partial ACL rupture [18]. These findings confirm results of our own cohort, which were previously published [1, 9]. However, there is no additional information about the degree of partial ACL tears available and overall just limited literature exists regarding the definition and classification of partial ACL tears [4].

Furthermore, all currently available studies concerning the use of PRP products to enhance ACL healing in partial ACL ruptures are characterized by a small cohort of patients and are limited to clinical and scoring results. Objective functional outcome measurements, such as functional performance tests, have not been published in context of outcome evaluation after partial ACL tears up to now; although, they are known to enable the physician to objectively assess the patients' knee function and ability to tolerate the daily physical demands in work and sport [25, 26]. The functional performance test mainly consists of two components, the quantity of movement and the quality of movement [27]. Both components are important factors for the assessment of rehabilitation quality as well as preventing ACL recurrent injury or treatment failure [27]. In this study the quantity and quality of movement were evaluated using a test battery including drop jump, side-hop, and quick feet as well as one- and two-leg stability tests. All of these tests showed promising results in all included patients. Also, regarding the different grades of partial ACL rupture an outcome depending on injury severity was documented in the drop jump, side-hop as well as one- and two-leg stability tests. Overall, particularly in the drop-jump test as well as in the side-hop test a clear benefit in comparison to the ACL reconstruction was detected [28, 29]. Bell et al. performed the drop-jump test in 29 patients after ACL reconstruction and 27 healthy patients. Overall, the results after intraligament ACP application and healing response technique almost conform to the results of the healthy group [28]. Similar results were shown for the side-hop test evaluated by Itoh et al. [29]. Comparing chronic ACL deficient patients with a healthy control group, the healthy control group achieved good results like the intraligament ACP/healing response technique cohort, whereas in the ACL deficient group inferior results were detected [29]. Hildebrandt et al. evaluated the one- and two-leg stability as well as the quick-feet tests in a healthy control group as a reference group [30]. In the one-leg stability test no significant differences were detected after ACP application and healing response technique as also seen in the reference group tested by Hildebrandt et al. [30]. Overall, in the one- and two-leg stability test as well as quick-feet test the intraligament ACP/healing response technique group was inferior to the healthy control group of Hildebrandt et al. These differences might be explained by the obvious younger and healthy study population assessed by Hildebrandt et al. However, further studies also determined prolonged stabilizing deficits in patients after ACL reconstruction, for example, in comparison to a healthy control even 2.5 years after surgery [31–35].

The clinical follow-up examination as well as postoperative scoring results are in accordance with results of the functional performance tests. In the included patients good anteroposterior knee stability with a firm endpoint in the Lachman test and no positive pivot shift phenomenon were detected. Rolimeter testing showed a significant improvement of the antero-postero translation of the knee joint after intraligament application of ACP and healing response in comparison to the preinjury status in all included patients (p = 0.001). Likewise, the functional scores correlate with the good results of the clinical examination and functional performance tests. They are comparable with previous data of the use of PRP products in partial ACL rupture as well as after ACL bundle reconstruction technique [1, 5, 6, 36].

Overall, the described technique for the treatment of partial ACL tears enables the patients to return to sport already after a short rehabilitation period. Training started after a mean of 12.8 weeks (SD 7.2 weeks) and the preinjury level of sport activity was achieved after a mean of 5.8 months (SD 3.6 months) in 71.1% of the reviewed patients. Regarding all included patients 85.8% (SD 19.0%) of the initial sportive capacity could be restored after intraligament ACP application and healing response technique. Here, especially in case of partial ACL tears grade 1 and 2 return to training (9.8 weeks, SD 4.8 weeks) and particularly to sport (4.9 months, SD 3.4 months) was accelerated and the return to preinjury sport level (88.9%) increased. These results correlate with the promising results previously published after the use of PRP products [1, 9, 18] and showed a clear advantage for this technique over both conservative ACL therapy [18, 36–38] as well as ACL reconstruction in recreational athletes as also reviewed in this study [39, 40].

Nevertheless, there are few limitations in this study, which have to be addressed in further studies. Due to the retrospective study design, no preoperative functional performance and scoring results are available. This study also lacks a comparative control group with serial MRI analysis. Furthermore, studies with higher numbers of participating patients are required to detect significant differences in the different degrees of injury severity and to other treatment options.

Thus, it can be concluded that the intraligament application of ACP in combination with the healing response technique is a promising treatment option for the therapy of partial ACL ruptures. Good functional performance results and concurrent good subjective clinical outcome results can be achieved by this technique at a midterm follow-up of averaged almost 3 years. Overall, the procedure is associated with a low failure rate and a high percentage of full recovery concerning the preinjury sport level. However, further high quality comparative and matched studies are needed to verify the qualities of the intraligament ACP application in combination with the healing response technique in comparison to the ACL reconstruction and to detect significant differences in the outcome of patients classified according to the grading system to develop a reliable treatment algorithm.

## Figures and Tables

**Table 1:** Grading system of partial ACL ruptures.

Grade	Definition
1	intact ACL sheet with haemorrhage of the synovial ACL sheet

2	ruptured synovial ACL sheet without extrusion of ACL tissue

3	ruptured synovial ACL sheet with extrusion of ACL tissue

4	partial rupture of one ACL bundle with 25-50% remaining ACL structure cave: in case of a two bundle partial ACL rupture, the percentage of the more injured bundle is used for classification

5	partial rupture of one ACL bundle with 10-25% remaining ACL structure cave: in case of a two bundle partial ACL rupture, the percentage of the more injured bundle is used for classification

**Table 2:** Epidemiological data and injury pattern.

	**all included patients** **n = 38**	Grade I / II n = 9	Grade III n = 23	Grade IV / V n = 6	failure n = 4
follow-up [months, mean (SD)]	**33.0 (17.4)**	38.7 (18.3)	30.5 (18.6)	34.0 (9.6)	-

age [years, mean (SD)]	**42.8 (13.5)**	39.1 (14.6)	43.7 (14.0)	45.2 (10.8)	26.8 (8.6)

gender [%; male/ female]	**44.7/55.3**	44.4/55.6	47.8/52.2	33.3/66.6	50/50

index side [%; right/ left]	**65.8/34.2**	88.9/11.1	60.9/39.1	50.0/50.0	50/50

dominant side [%; right/ left]	**78.9/21.1**	77.8/22.2	60.9/39.1	100/0	75/25

concomitant injury [%]	**55.3**	33.3	60.9	66.7	75.0

meniscus [%]	**80.9**	66.7	47.8	66.7	75.0

collateral ligament [%]	**47.6**	33.3	39.1	0	0

sports associated injury	**84.2**	88.9	65.2	100	100

football [%]	**15.6**	12.5	20.0	16.7	25

ski [%]	**62.5**	62.5	73.3	66.7	50

mountain biking [%]	**3.1**	12.5	0	0	0

volleyball [%]	**6.25**	12.51	6.7	0	25

kung-fu [%]	**3.1**	0	0	16.7	0

pre-injury sport level					

recreational [%]	**55.3**	55.6	52.2	66.7	25

amateur [%]	**42.1**	33.3	47.8	33.3	75

professional [%]	**2.6**	11.1	0	0	0

**Table 3:** Return to sport data.

	**all included patients**	Grade I / II	Grade III	Grade IV / V
return to …	**mean (SD)**	mean (SD)	mean (SD)	mean (SD)

training [weeks]	**12.8 (7.2)**	9.8 (4.8)	14.4 (7.6)	12.3 (8.3)

sports [months]	**5.8 (3.6)**	4.9 (3.4)	6.0 (2.2)	6.8 (5.8)

pre-injury sport level [%]	**71.1**	88.9	43.5	100

subjective regain of sport level [%]	**85.8 (19.0)**	86.9 (18.5)	82.8 (21.3)	94.2 (8.0)

**Table 4:** Clinical outcome data.

	**all included patients**	Grade I / II	Grade III	Grade IV / V
ROM deficit [%]	**10.5**	0	13.0	16.7

LM test				

negative [%]	**100**	100	100	100

positive [%]	**0**	0	0	0

pivot shift				

negative [%]	**100**	100	100	100

positive [%]	**0**	0	0	0

rolimeter test				

pre-operative [mm]	**1.9 (SD 1.4)**	1.7 (SD 1.5)	1.7 (SD 1.4)	3.2 (SD 1.0)

post-operative [mm]	**0.6 (SD 1.8)**	0.6 (SD 1.7)	1.2 (SD 1.8)	0.2 (SD 1.2)

p-value	**0.001** **∗**	0.01*∗*	0.4	0.0007*∗*

[*∗*] = sign for significance; significance level < 0.05.

**Table 5:** Postoperative outcome scoring.

score	**all included patients**		Grade I / II	Grade III	Grade IV / V
	**mean (SD)**		mean (SD)	mean (SD)	mean (SD)
IKDC subjective	**83.2 (14.5)**		85.6 (18.4)	80.1 (13.8)	89.7 (8.1)
Lysholm	**85.5 (15.5)**		83.2 (26.6)	85.4 (10.3)	89.3 (8.5)
Tegner	**4.7 (1.7)**		5.1 (2.1)	4.6 (1.6)	4.6 (1.9)
Cincinnati	**85.4 (15.5)**		81.6 (23.5)	86.4 (11.7)	90.0 (8.9)
Marx	**4.8 (4.4)**		7.0 (4.4)	3.5 (3.8)	1.7 (0.8)

IKDC objective		**[**%**]**	[%]	[%]	[%]
	**A**	**59.4**	62.5	61.1	50.0
	**B**	**28.1**	25.0	27.8	33.3
	**C**	**12.5**	12.5	11.1	16.7
	**D**	**0**	0	0	0

**Table 6:** Functional performance tests.

functional performance test	**all included patients**	Grade I / II	Grade III	Grade IV / V
	**mean (SD)**	mean (SD)	mean (SD)	mean (SD)

**drop jump test**				

index side [points]	**7.4 (1.2)**	7.9 (1.0)	7.3 (1.3)	6.8 (1.1)

healthy side [points]	**7.5 (1.3)**	7.5 (1.1)	7.7 (1.3)	6.8 (1.6)

p-value	**n.s.**	n.s.	n.s.	n.s.

**side-hop-test**				

(1) trial [sec]	**0.2 (1.9)**	0.7 (0.9)	0.6 (1.8)	-1.8 (1.3)

(2) trial [sec]	**0.6 (1.6)**	0.4 (1.0)	0.7 (1.8)	0.9 (1.8)

**two-leg-stability test**				

(1) trial [sec]	**3.5 (0.8)**	3.2 (0.6)	3.5 (0.8)	3.8 (0.7)

(2) trial [sec]	**3.1 (0.8)**	3.0 (0.3)	3.1 (1.0)	3.5 (0.8)

**one-leg-stability test**				

index side				

(1) trial [sec]	**2.9 (0.9)**	2.8 (0.9)	2.9 (1.0)	3.1 (0.6)

(2) trial [sec]	**2.9 (0.7)**	2.6 (0.5)	2.9 (0.8)	3.2 (0.9)

healthy side				

(1) trial [sec]	**3.0 (0.8)**	2.9 (0.8)	3.0 (0.8)	3.3 (0.9)

(2) trial [sec]	**2.8 (0.8)**	2.7 (0.5)	2.8 (0.9)	3.1 (0.7)

p-value (index vs healthy side)	**n.s.**	n.s.	n.s.	n.s.

**quick-feet-test**				

[sec]	**12.0 (3.4)**	10.7 (1.6)	12.6 (3.9)	11.8 (2.7)

n.s. = not significant according the level of significance > 0.05.

## Data Availability

The datasets generated and analysed during the current study are not publicly available but are available from the corresponding author on reasonable request.
